# Local Treatment with Lactate Prevents Intestinal Inflammation in the TNBS-Induced Colitis Model

**DOI:** 10.3389/fimmu.2016.00651

**Published:** 2016-12-27

**Authors:** Carolina Iraporda, David E. Romanin, Ana A. Bengoa, Agustina J. Errea, Delphine Cayet, Benoit Foligné, Jean-Claude Sirard, Graciela L. Garrote, Analía G. Abraham, Martín Rumbo

**Affiliations:** ^1^Centro de Investigación y Desarrollo en Criotecnología de Alimentos (CIDCA, UNLP-CONICET-CIC.PBA), La Plata, Argentina; ^2^Instituto de Estudios Inmunológicos y Fisopatológicos (IIFP, UNLP-CONICET), La Plata, Argentina; ^3^CNRS, INSERM, CHU Lille, Institut Pasteur de Lille, U1019 – UMR 8204 – CIIL – Center for Infection and Immunity of Lille, University of Lille, Lille, France; ^4^Área Bioquímica y Control de Alimentos, Facultad de Ciencias Exactas, UNLP, La Plata, Argentina

**Keywords:** innate immunity, lactate, TNBS-induced colitis, flagellin, immunomodulation

## Abstract

Lactate has long been considered as a metabolic by-product of cells. Recently, this view has been changed by the observation that lactate can act as a signaling molecule and regulates critical functions of the immune system. We previously identified lactate as the component responsible for the modulation of innate immune epithelial response of fermented milk supernatants *in vitro*. We have also shown that lactate downregulates proinflammatory responses of macrophages and dendritic cells. So far, *in vivo* effects of lactate on intestinal inflammation have not been reported. We evaluated the effect of intrarectal administration of lactate in a murine model of colitis induced by 2,4,6-trinitrobenzenesulfonic acid (TNBS). The increase in lactate concentration in colon promoted protective effects against TNBS-induced colitis preventing histopathological damage, as well as bacterial translocation and rise of IL-6 levels in serum. Using intestinal epithelial reporter cells, we found that flagellin treatment induced reporter gene expression, which was abrogated by lactate treatment as well as by glycolysis inhibitors. Furthermore, lactate treatment modulated glucose uptake, indicating that high levels of extracellular lactate can impair metabolic reprograming induced by proinflammatory activation. These results suggest that lactate could be a potential beneficial microbiota metabolite and may constitute an overlooked effector with modulatory properties.

## Introduction

Inflammatory bowel disease (IBD) involves a group of chronic, inflammatory disorders of the gastrointestinal tract, including Crohn’s disease and ulcerative colitis, affecting people of all ages including the pediatric population. The etiology of IBD is still unknown but is thought to be due to a combination of genetic, microbial, immunological, and environmental factors that result in an abnormal and excessive immune response against commensal microbiota (1). The intestinal microbiota profoundly regulates the host immune function under physiological conditions and is likely the most important environmental factor in IBD as the target of the inflammatory response (2).

Dysbiosis or a lack of specific bacteria with anti-inflammatory properties may be responsible for gut inflammation (3–6). Although the molecular mechanisms of host–microbiota interactions are still not fully elucidated, manipulation of microbiota by probiotics or prebiotics is becoming increasingly recognized as a therapeutic option, for the treatment of the dysfunction or inflammation of the intestinal tract (7). The metabolic output of the modification of gut microbiota is the production of different profiles of short chain fatty acids (SCFA) such as butyrate, propionate, and acetate, and these metabolites are of relevance in the modulation of key signaling pathways involved in the inflammation of the gastrointestinal mucosa (7–9).

The impact of probiotic bacteria on intestinal health with the aim to prevent IBD or improve its treatment has been studied (10–12), as well as it has been shown that metabolites present in the supernatants of fermented dairy products can exert a protective effect *ex vivo* on intestinal mucosa exposed to inflammatory insults (13).

Lactate is the main metabolite of many fermented products and can also be generated *in situ* on the intestinal mucosa. Although lactate has been known to biochemists for over 200 years, it has been considered as a mere intermediate of carbon metabolite with specific organoleptic/antimicrobial properties rather than a bioactive molecule. Recently, lactate has been rediscovered as an active signaling metabolite in multiple fields of biology and medicine (14). Lactate mediates signaling pathways on several cell types, including production of pro- and anti-inflammatory mediators by T cells and macrophages and migratory changes and metabolic adaptation in T cells, endothelial cells, and neurons. Intracellular lactate can directly bind to proteins, influence the redox state *via* the lactate dehydrogenase reaction, stabilize hypoxia inducible factor-1, induce reactive oxygen species, and act as an inhibitor of glucose breakdown (15). The occurrence of these effects might depend on the cell type. Hoque et al. (16) demonstrated that administration of lactate reduced inflammation and organ injury in mice with immune hepatitis (16). Moreover, besides immunomodulation, Okada et al. (17) showed that luminal lactate-stimulated enterocyte proliferation in a murine model of hunger feedback, contributing to maintain intestinal barrier function (17). We have recently shown that lactate abrogates TLR and IL-1β dependent NF-κB activation of intestinal epithelial cells (18) and can regulate critical functions of several key players of the immune system such as macrophages and dendritic cells (19). In order to determine if the immunomodulatory capacity of lactate operates *in vivo*, the present work evaluated the effect of lactate in innate-driven murine model of colitis.

## Materials and Methods

### Chemicals and Reagents

Different chemical reagents used 2,4,6-trinitrobenzenesulfonic acid (TNBS), 2 deoxyglucose (2DO), sodium oxamate, sodium 3-bromopyruvate (3BrPA) were purchased to Sigma Chemicals. dl-lactic acid (J. T. Baker) was employed. Flagellin was purified from *Salmonella*, detoxified, and controlled as previously described (20). Other proinflammatory stimulators, such as human interleukin-1β (IL-1β) and tumor necrosis factor (TNF), were purchased from R&D Systems (Minneapolis, MN, USA).

### Animals

Male BALB/c AnN, 6 weeks old mice with weight over 20 g were purchased from Faculty of Science Veterinary from National University of La Plata, Argentina. The animals kept in polypropylene cages were maintained under standard conditions. The experimental protocols were approved by the Animal Ethics Committee of Faculty of Exact Sciences, National University of La Plata, Argentina (Approval No 011-01-15). Before conducting experiments, animals were acclimatized to animal facility conditions for 7 days.

### Treatment and Induction of Experimental Colitis Using TNBS

Procedure was performed as previously described (21). Briefly, mice randomly divided into four groups were instilled with PBS (200 µL) (two groups) and with lactate solution in PBS 200 mM (200 µL) (two groups) by intrarectal route. Two hours post-administration, experimental colitis was induced by intrarectal instillation of 0.5 mg TNBS (SIGMA-Aldrich, USA) in ethanol 50% (v/v). Control animals were instilled with ethanol 50% (v/v) in distilled water. Enemas were gently instilled through a polyurethane catheter (18 G) inserted into the colon 4 cm proximally to the anal verge, and mice were held thereafter in a head-down position for 30 s. The weight of each mouse was determined and blood sampled at the beginning of the experiment and at 24 and 48 h. After 48 h, animals were sacrificed by cervical dislocation; colon tissues were collected for histological analysis (hematoxylin and eosin staining); and livers were aseptically taken to determine microbial translocation.

### Serum IL-6 Determination

Blood was collected by submandibular bleeding and serum was isolated. Serum IL-6 determination was performed using BD Bioscience OptEIATM Mouse IL-6 ELISA Kit (Franklin Lakes, NJ, USA), according to manufacturer instructions.

### Assessment of Colonic Epithelial Damage and Inflammation

Histopathological damage was determined following the criteria described previously (21). This system records two separate scores evaluating epithelial damage and infiltration. Briefly, the epithelial damage was scored as 0 for none, 1 for a minimal loss of goblet cells, 2 for extensive loss of goblet cells, 3 for a minimal loss of crypts and extensive loss of goblet cells, and 4 points for extensive loss of crypts; the infiltration was scored as 0 for none, 1 for an infiltrate around crypts bases, 2 for an infiltrate in *muscularis mucosa*, 3 for extensive infiltrate in *muscularis mucosa* with edema, and 4 points for the infiltration of submucosa. Preparations were assessed double blind, and the histopathological activity index was calculated as the sum of the epithelial damage and the infiltration score, ranging between 0 and 8 points from unaffected to severe colitis.

### Microbial Translocation

Portions of liver were aseptically collected and placed in a sterile tube with a volume of BHI broth (Oxoid, England) in order to obtain 1 g organ/10 mL. These suspensions were homogenized, enriched in total viable bacteria by incubation 24 h at 37°C and used to inoculate BHI agar plates. Translocation of bacteria was defined by growth of microorganism on plates after 48–72 h of incubation at 37°C.

### Cell Culture and CCL20:LUC Reporter Assay

Caco-2 cells stably transfected with a luciferase reporter construction under the control of the chemokine-ligand-20 (CCL20) promoter (Caco-2-*CCL20:LUC*) have been previously described (20). The cells were routinely grown in Dulbecco’s Modified Eagle’s Minimum Essential Medium (DMEM, GIBCO BRL Life Technologies, Rockville, MD, USA); supplemented with 15% (v/v) heat-inactivated (30 min, 60°C) fetal-bovine serum (FBS, PAA, GE Healthcare Bio-Sciences Corp., USA), 1% (v/v) non-essential amino acids (GIBCO BRL Life Technologies, Rockville, MD, USA) and the following antibiotics (Parafarm, Saporiti SACIFIA, Buenos Aires, Argentina): penicillin (12 IU/mL), streptomycin (12 µg/mL), and gentamicin (50 µg/mL). Caco-2-*CCL20:LUC* cells were used at 24 h post-confluence after 8 days of culture at subculture passages between 12 and 22 from the original stocks. All experiments were performed in serum-free medium.

Confluent Caco-2-*CCL20:LUC* cells cultured in 48-well plates were treated for 30 min with different concentrations of lactate pH 7.4 or different solutions of glycolysis inhibitors. The cells were then exposed to stimulation by flagellin (1 µg/mL), Il-1β (10 ng/mL), or TNF-α (100 ng/mL), during 6 h at 37°C in an atmosphere of 5% CO_2_—95% air. A basal condition without any treatment was included as a control lacking stimulation; while flagellin, TNF-α, or IL1-β was added to cell that did not receive any treatment as control of 100% of induction of the proinflammatory response. The cells were next lysed with lysis Buffer (Promega, Madison, WI, USA), and luciferase activity was evaluated using the Luciferase Assay Kit (Promega, Madison, WI, USA) following manufacturer’s instructions and measured in a luminometer (Luminoskan TL Plus). Luminescence was normalized to the stimulated control cells and expressed as a percentage of the normalized average luminescence (% normalized luciferase activity) ± SD from at least three independent experiments.

### Cytotoxicity Assay

As a method of assessing treatment-induced cytotoxicity, mitochondrial activity was evaluated employing commercial kit CellTiter 96^®^ AQueous One Solution Cell Proliferation Assay (Promega, Madison, WI, USA) following manufacturer’s instruction.

### Glucose Consumption by Epithelial Cells against TLR5 Agonist Stimulation

Confluent Caco-2/TC-7 epithelial cells cultured in 48-well plates were incubated at 37°C, in controlled atmosphere 5% CO_2_ in DMEM containing initially 2 g/L glucose. Glucose uptake was determined in the culture medium employing a commercial enzymatic kit (Wiener lab, Rosario, Argentina). Samples were taken after 3, 6, 15, and 24 h of incubation either in basal condition, stimulated with flagellin with and without lactate 100 mM in the culture medium.

### Statistical Analysis

The results are expressed as mean ± SD. Data analysis was performed using Graph Pad Prism version 5.01 for Windows (GraphPad Software, CA, USA). Analyses of variance followed by Dunnet Test or Bonferroni Test were applied. A *p*-value <0.05 indicated a significant difference.

## Results

### Lactate Treatment Prevents Tissue Inflammation, Early IL-6 Production, and Bacterial Translocation in a TNBS-Induced Colitis Model

**Figure 1:**
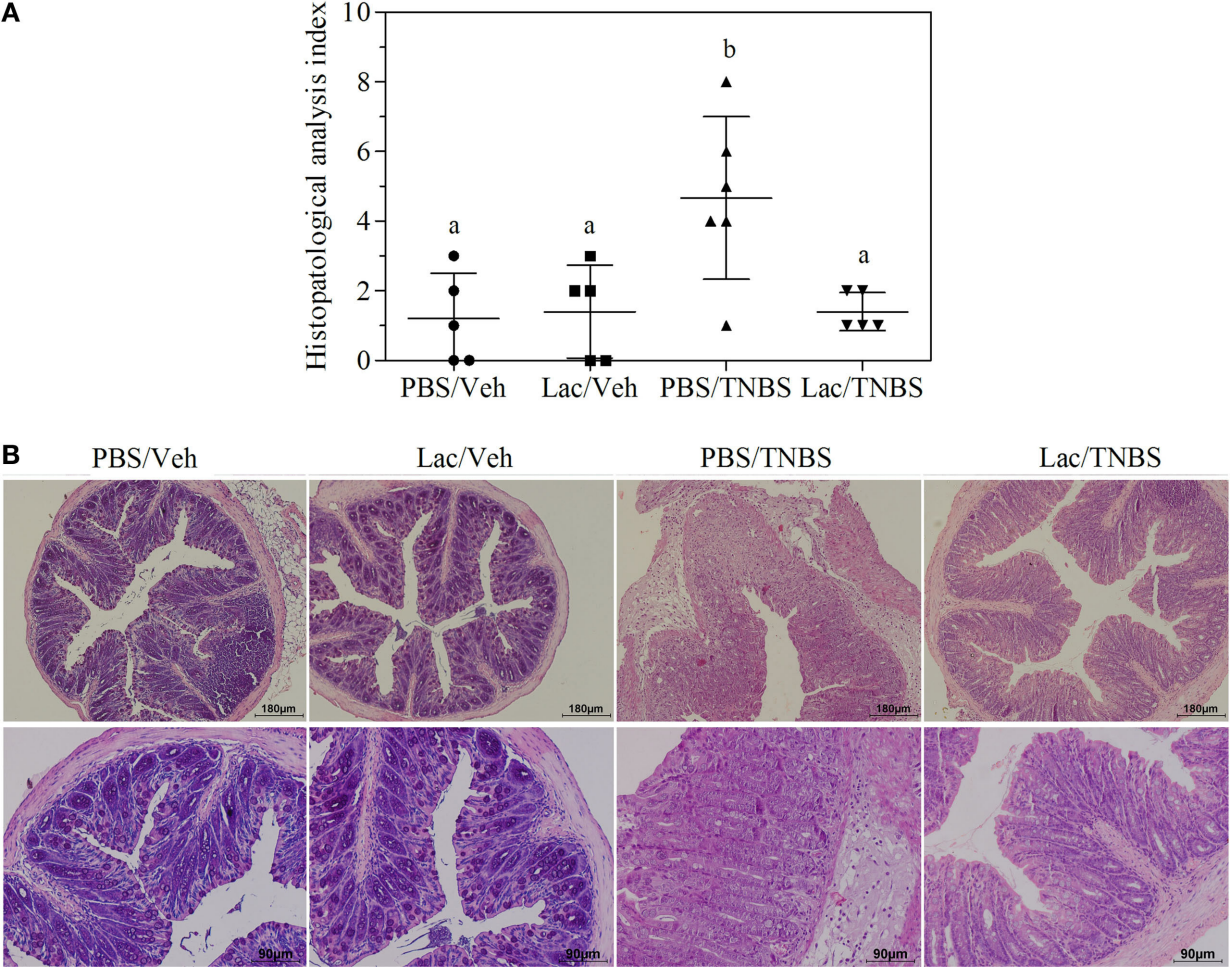
**Rectal administration of lactate protects animals against damage in TNBS acute colitis model**. Mice were treated with lactate or PBS (i.r.) 2 h before TNBS or vehicle instillation and 48 h afterward tissue was collected for histopathological analysis. In all cases, groups of at least five mice were used. Results from a representative experiment out of five are shown. **(A)** Histopathological activity index assigned to different experimental groups. Different letter indicates significant differences with *p* < 0.05. **(B)** Photomicrograph of H&E-stained cross section (×100 top line and ×200 bottom line) of distal colon of a representative mouse of each experimental groups.

To address the *in vivo* immunomodulatory capacity of lactate, we evaluated the capacity to protect mice from colitis induced by intrarectal administration of TNBS. During the experiment, we compared the development of TNBS-induced colitis in mice that received intrarectal administration of lactate 200 mM or PBS as control. Such administration guaranteed lactate contact with intestinal cells exposed to the TNBS. The intrarectal administration of PBS or lactate followed by vehicle administration did not induce any significant changes of animal weight. In contrast, the rectal administration of TNBS causes progressive weight loss reaching up to 15% of the initial weight at 48 h. In both TNBS-treated groups (PBS/TNBS and Lactate/TNBS), a significant weight loss was observed (Figure S1 in Supplementary Material). Although the differences were not significant, the weight loss was lower in lactate/TNBS than in PBS/TNBS (10 versus 15%). Histological features of colitis were observed in the PBS/TNBS group as determined by epithelial damage, loss of goblet cells, edema, and infiltration of immune cells, leading to a pathology index of 4.67 ± 2.33 (Figure 1). In contrast, the group of mice pretreated with lactate (lactate/TNBS) showed significant protection from TNBS-induced inflammation, with lack of epithelial damage and minimal edema. Indeed, the histological sections were similar to the control groups that did not receive TNBS (PBS/vehicle and lactate/vehicle). The histopathology index of 1.40 ± 0.54 was significantly different from that of PBS/TNBS group (*p* = 0.039) (Figures 1A,B). This was in concordance with a clear better behavior of lactate-treated animals, indicating that lactate treatment prevents intestinal inflammation in the TNBS colitis model.

**Figure 2:**
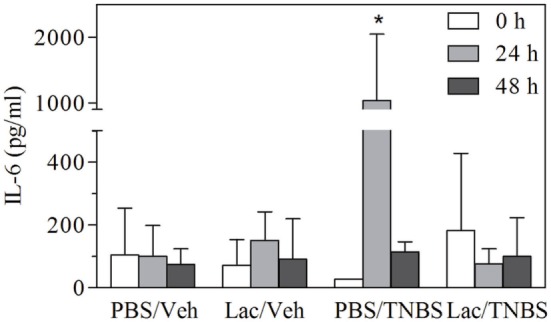
**Rectal administration of lactate protects animals against early IL-6 production**. Serum was obtained from animals treated as in Figure 1, and levels of IL-6 were measured by ELISA. Serum levels of IL-6 (pg/ml) (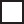) before (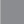) 24 h (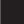) 48 h, after TNBS or vehicle administration. Results from a representative experiment out of five are shown, expressed as the mean ± SD, *indicates significant difference with *p* < 0.05 respect to its corresponding control.

Inflammation is associated with the production of various inflammatory mediators, primarily cytokines that are key players in the innate and adaptive immune responses. Levels of circulating IL-6 were determined in the different experimental groups before and 24 or 48 h after instillation (Figure 2). IL-6 levels were significantly increased 24 h after treatment with TNBS in control group but decreased to the baseline at 48 h. In contrast, lactate treatment abolished the production of circulating IL-6 at 24 h, resulting in levels similar to control group. These results indicate that lactate pretreatment also prevents systemic alteration induced by TNBS treatment.

2,4,6-trinitrobenzenesulfonic acid is known to disrupt the mucosal barrier function by interacting with surface-active phospholipids of the colonic mucosa, a process that is evidenced by microbial translocation. We assessed the disruption by measuring the presence of bacteria into the liver. Our data demonstrated bacterial translocation four out of six animals in the PBS/TNBS group (Table 1). On the contrary, we did not find any bacteria in liver of animals pretreated with lactate and exposed to TNBS. These results were similar to the groups of mice that received vehicle (PBS/vehicle and lactate/vehicle) and did not experimented disruption of the mucosal barrier. Overall, our results show that luminal lactate could prevent bacterial translocation and reduce tissue inflammation induced by TNBS.

**Table 1:** **Microbial translocation to liver observed 48 h after 2,4,6-trinitrobenzenesulfonic acid (TNBS)-induced colitis**.

Treatment	Animals with positive translocation/total animals in the group
PBS/VEH	0/5
LAC/VEH	0/5
PBS/TNBS	4/6
LAC/TNBS	0/5

*Results from a representative experiment out of five are shown*.

### Lactate Downregulates Proinflammatory Response in Intestinal Epithelial Cells and Induces Metabolic Changes

**Figure 3:**
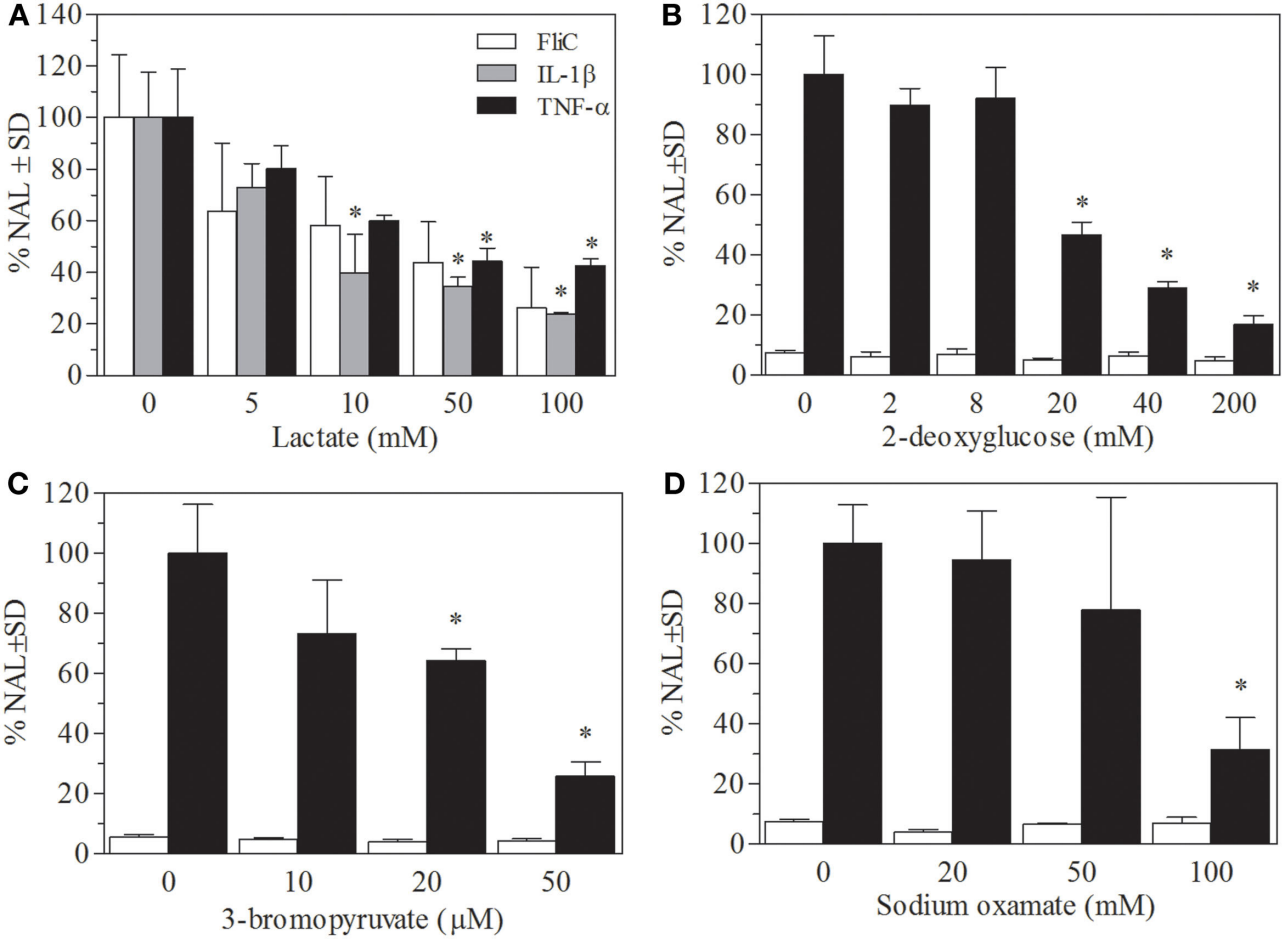
**Lactate pretreatment as well as glycolysis inhibition leads to downregulation of inflammatory response in Caco-2-*CCL20:LUC* cells**. Reporter cells were stimulated with IL1-β (10 ng/mL), tumor necrosis factor (TNF)-α (100 ng/mL), or flagellin (1 µg/mL), after pretreatment with different concentrations of lactate. **(A)** Results are expressed as normalized luciferase activity, using the levels of stimulated cells in absence of lactate as 100% of activation. The Caco-2-*CCL20:LUC* cells pretreated with solutions of glycolysis inhibitors **(B)** 2DG (mM) **(C)** 3-bromopyruvate (μm) **(D)** Oxamate (mM). Results shown are the mean and SEM from independent triplicates. Results from a typical experiment out of at least three are depicted. 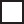 Non-stimulated and 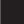 stimulated Flic. *Indicates a significant difference from the cells without treatment and stimulated with flagellin, IL-1β, and TNF-α, respectively, with *p* < 0.05.

In order to unravel the mechanisms of the anti-inflammatory effect of lactate in mice, an *in vitro* assay in the intestinal epithelial cell line Caco-2-*CCL20:LUC* that enables the monitoring of proinflammatory activation was used. In concordance to previous reports, pretreatment of Caco-2-*CCL20:LUC* cells with lactate produced a significant decrease of luciferase activity induced by various proinflammatory stimuli, i.e., flagellin (the TLR5 agonist), the cytokines IL1-β, and TNF (Figure 3A). In all stimulation conditions, a similar pattern of downregulation of the proinflammatory signaling was observed. For instance, exposure to concentrations of lactate of 5 mM or higher elicited a significant decrease of IL1-β-induced activation. These inhibitory effects of lactate were increased in a dose-dependent manner.

We have previously observed that lactate treatment abrogates enhanced glycolysis in TLR-stimulated macrophages, which correlates with its activity as modulator of innate response (22), Caco-2-*CCL20:LUC* reporter cell line was utilized to evaluate if the effects of lactate on epithelial cells could be related to metabolic changes.

Treatment of cells during 6 h with glycolysis inhibitors such as 2DO or 3BrPA (competitive inhibitors of hexokinase) and oxamate (inhibitor of lactate dehydrogenase) in different concentrations did not affect luciferase activity in non-stimulated condition. Luciferase activity induced by flagellin was significantly lower in cells pretreated with glycolysis inhibitors, and this effect was dose-dependent (Figures 3B–D). Cell viability was not affected by 6 h incubation with glycolysis inhibitors, showing an enzymatic activity on MTT reduction over 85% in all cases (not shown).

**Figure 4:**
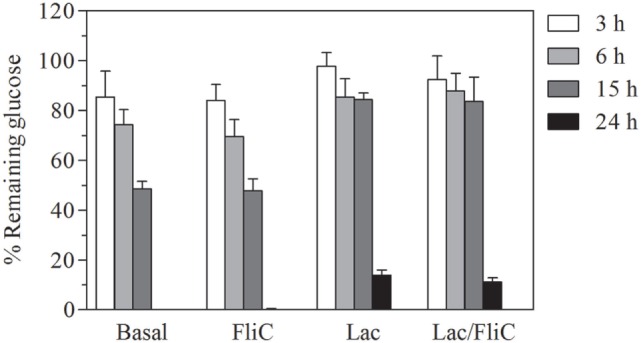
**Lactate treatment decreases glucose uptake of Caco-2/TC-7 cells either in basal as well as stimulated conditions**. Percentage of glucose remaining in culture medium of Caco-2/TC-7 cells either with lactate 100 mM or not, non-stimulated and stimulated with flagellin (1 µg/mL), incubated for (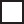) 3, (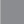) 6, (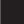) 15, and (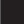) 24 h.

We observed that Caco-2 intestinal epithelial cells decreased their rate of glucose consumption in the presence of lactate either in basal as well as with flagellin conditions. This could be associated with an inhibition of glycolysis in presence of lactate (Figure 4). Overall, these results indicate that lactate modulation of epithelial response, correlates with its capacity to alter glycolytic activity, which alters the capacity to trigger the effectors of innate response activation.

## Discussion

Although the etiology of IBD is still unknown, increasing evidence shows that IBD may involve in genetically susceptible individuals a dysregulation of their immune response to resident microbiota (23). It is now widely accepted that a misbalanced gut ecosystem also plays an important role other pathologies of the gastrointestinal tract (24).

Several therapeutic strategies proposed to reduce the symptoms of IBD are based on the use of anti-inflammatory drugs (such as corticoids, 5-aminosalicylic acid, and anti-TNF-α antibodies), all having marked long-term side effects. Other proposed treatments are based on microbiota-based dietary interventions, either by the use of probiotics or prebiotics (25). Therapeutic administration of probiotic species such as *Bifidobacterium* spp., *Lactobacillus* spp., or *Propionibacterium* has been shown to have protective effects on IBD models through the production of anti-inflammatory metabolites (26, 27). Almost two decades ago, proof of concept in clinical studies demonstrated the efficacy of SCFAs-based treatments in IBD, specifically in ulcerative colitis, treating the inflamed region using a mixture of SCFAs (acetate 80 mM, butyrate 40 mM and propionate 30 mM) enemas (28–30). In the recent years, therapeutic strategies related to intestinal SCFAs to manage IBDs have renewed interest based on studies from either animal models or intestinal metabolomic/microbiota analysis on patients. Interventions in animal models resulting in increased exposure of intestinal tissue to specific SCFAs have shown protective effects in intestinal mucosa (31, 32) and new combined interventions with pharmaceuticals and oral SCFAs formulated to be released in large intestine have shown efficacy in patient management (33).

We have previously shown that lactate can downregulate the proinflammatory responses of immune cells such as macrophages and dendritic cells, as well as those of mucosal structural cells like intestinal epithelial cells (18, 19). The diverse effects of lactic acid on various immune cells suggest that lactic acid or lactate may influence widely used signaling pathways. Indeed, both molecules have been demonstrated to influence several MAP kinases, NFkB signaling, or the PI3K/AKT pathway (15, 34). Aiming to analyze effects of lactate on inflammation *in vivo*, in a proof of concept experimental design, we found that pretreatment with lactate 200 mM modulates the epithelial damage and infiltration induced by TNBS. This effect was not observed when lactate 200 mM was administered in drinking water, on account of low lactate levels measured in distal colon (not shown). This reduced content could be due to either intestinal absorption by enterocytes, lactate consumption by microbiota, or both. To have protective effect, lactate luminal levels should be high, such as those reached by intrarectal administration. TNBS i.r. administration has been used as model for innate-driven intestinal inflammation due to epithelial damage and increased access of microbial-derived molecules to the immune cells in the lamina propria compartment (35). The use of the TNBS model that produce an acute local activation of inflammatory response allowed us to evaluate the local effect of lactate after a short term exposure, upon i.r intervention. Due to experimental design, contribution of microbiota, other fermentation metabolites, or other microbial products to the anti-inflammatory effect is expected to be low, indicating that is lactate the main responsible for the modulation observed.

Using different strategies, several authors showed that prevention of inflammation in the TNBS model is usually correlated with lower bacterial translocation from the gut to mesenteric lymph nodes and systemic compartment (36–40). In coincidence with these results, we have shown that lactate administration protects against microbial translocation to the liver in animals treated with TNBS and the increase in lactate concentration in colon alleviates TNBS-induced colitis. Furthermore, lactate treatment also prevented serum rise of levels of IL6 (Figure 2), in coincidence with our previous observations that lactate pretreatment abrogates NFkB activation and proinflammatory gene expression such as IL12, IL1β, or IL6 (18, 19). Some proinflammatory cytokines that may be modulated in this way, such as IL1b and IL18, have also the capacity to trigger epithelial renewal and reinforce barrier function (41). Nevertheless, in our system, the overall effect of lactate is to promote tissue protection as appreciated by hystopathological analysis (Figure 1).

There are several possible non-mutually exclusive mechanisms that may explain the capacity of lactate to prevent inflammation in our model. We have recently shown that lactate, as other SCFAs, may prevent TLR-mediated activation of macrophage and dendritic cells (19). Several reports indicate that the blockage of macrophage activation can modulate colonic inflammation in different acute models; Du et al. (42) have shown that targeting intestinal macrophages with gadolinium chloride block colitis in a TNBS model (42). Furthermore, several treatments targeting intestinal macrophage activation, using miRNAs or modulation of specific GPCRs, can also modulate colitis in TNBS model (43, 44). Besides, previous studies have shown that lactate can modulate innate activation of intestinal epithelial cells (18, 19). Since epithelial cells can also contribute to the amplification of inflammation in the TNBS model, this could be another possible cellular target that explains the bioactive properties of lactate. In line with this possibility, Cheng et al. have shown that targeting intestinal epithelial cells may reduce colitis in IBD models (45).

Beyond the cellular target of lactate, there are also several mechanisms that may account for its activity. We have recently shown that lactate impairs macrophage metabolic reprograming after LPS activation in a GPR81-independent manner (22), which has also been associated with blunting the proinflammatory cytokine response (46). In accordance with these results, Selleri et al. (47) have shown that local increase of lactate in the environment of mesenchymal stromal cells shifts macrophage M1 activation toward the less inflammatory M2 (47). Colegio et al. (48) have shown also the blunting of M1 macrophage activation in the solid tumor environment due to high lactate production of Warburg metabolism of tumor cells (48). Kreutz and colleagues have shown that increase in extracellular lactate of macrophages impair proinflammatory activation by altering its capacity to rise its glycolitic flux, effect that is enhanced at low pH (34, 49). Inhibition of lactate efflux from macrophages blocks LPS-driven activation by a mechanism also associated to impairment of glycolytic reprograming of macrophage upon activation (50).

Metabolic reprograming upon proinflammatory activation of myeloid cells implicates enhanced glycolysis with low respiratory rate (51). In the case of macrophages, this implies high rate of urea cycle intermediates for the production of NO from arginine (46) and production of lipid metabolites from citrate. In the case of dendritic cells, metabolic reprograming supplies carbon from glycolysis to lipid metabolites, mainly to allow expansion of endoplasmic reticulum in order to facilitate antigen presentation (52). Although it is not clear that the extent of metabolic reprograming takes place in epithelial cells upon TLR activation, there are some reports that show similarities on macrophage and epithelial cell response to mediators of metabolic reprograming (53). Our results indicate that blocking of glycolysis impairs flagellin-induced CCL20 transcriptional activation observed in our reporter system (Figure 3). Furthermore, we observed less consumption of glucose in the presence of extracellular lactate (Figure 4). These results are consistent with the necessity of enhancement of glycolysis rate in epithelial cells for a full functional TLR response, as is the case of macrophages.

Although our experimental design was first aimed to confirm the bioactive properties of lactate observed *in vitro* in a preclinical model, it opens the possibility of using lactate in local treatments to modulate inflammation. Furthermore, it can be considered that local production of lactate by probiotic microorganisms that attach to the intestinal epithelium may also contribute to their protective capacity in inflammatory situations (14), providing alternative cues for selection of microorganisms to be used as complement in the management of IBDs.

## Conclusion

Results shown here were conclusive in relation to the effect of lactate at local level in a model of acute intestinal inflammation, contributing to a decrease in epithelial damage, signs of inflammation, and the secretion of proinflammatory cytokine IL-6, presenting a first approximation *in vivo* about the role of lactate in preventing intestinal inflammation.

Although several possibilities remain to be considered to explain the cellular and molecular mechanisms responsible for the observed effect, a correlation between impairment of glycolysis and proinflammatory activation of epithelial cells was observed, in coincidence with previous works in macrophages.

These results suggest that lactate could be a potential beneficial microbiota metabolite and may contribute to health-promoting properties on the intestinal mucosa.

## Author Contributions

CI performed experimental work, participated in the study design and conception and manuscript writing. AB, DR, AE, and DC performed experimental work, participated in study design. BF performed experimental work, participated in study design and manuscript writing. J-CS participated in study design, funding, and manuscript writing. GG, AA, and MR participated in study design and conception, funding, and manuscript writing.

## Conflict of Interest Statement

The authors declare that the research was conducted in the absence of any commercial or financial relationships that could be construed as a potential conflict of interest. The handling Editor declared a shared affiliation, though no other collaboration, with several of the authors and states that the process nevertheless met the standards of a fair and objective review.

## Supplementary Material

Figure S1 **Intrarectal administration of lactate protects animals against weight loss in 2,4,6-trinitrobenzenesulfonic acid (TNBS) acute colitis model**. Weight variation after 24 and 48 h of TNBS-induced colitis (% of initial weight). In all cases, groups of at least five mice were used. Results from a representative experiment out of five are shown. Different letter indicates significant differences with *p* < 0.05.
