# Supplementary figures and images for: Local Treatment with Lactate Prevents Intestinal Inflammation in the TNBS-Induced Colitis Model
Source: Front Immunol. 2016 Dec 27;7:651. doi: 10.3389/fimmu.2016.00651 (PMC5187354; doi:10.3389/fimmu.2016.00651)

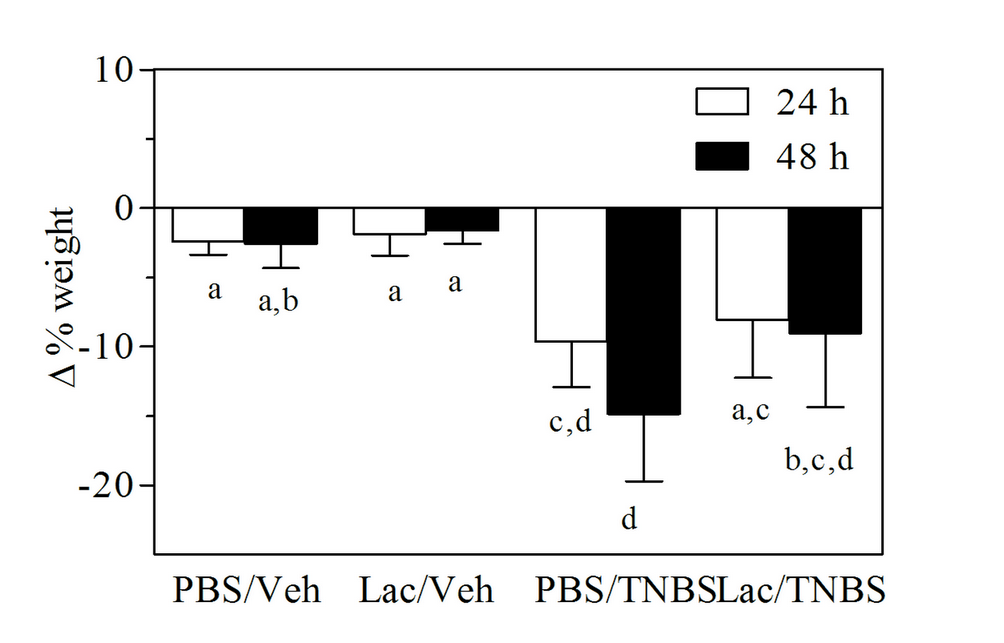

Supplement: Figure S1 — Intrarectal administration of lactate protects animals against weight loss in 2,4,6-trinitrobenzenesulfonic acid (TNBS) acute colitis model. Weight variation after 24 and 48 h of TNBS-induced colitis (% of initial weight). In all cases, groups of at least five mice were used. Results from a representative experiment out of five are shown. Different letter indicates significant differences with p < 0.05. [file Image_1.TIF]
